# Development of abstract mathematical reasoning: the case of algebra

**DOI:** 10.3389/fnhum.2014.00679

**Published:** 2014-09-02

**Authors:** Ana Susac, Andreja Bubic, Andrija Vrbanc, Maja Planinic

**Affiliations:** ^1^Department of Physics, Faculty of Science, University of ZagrebZagreb, Croatia; ^2^Chair for Psychology, Faculty of Humanities and Social Sciences, University of SplitSplit, Croatia

**Keywords:** mathematics, education, algebra, problem solving, cognitive development, abstract reasoning, concrete reasoning, strategy

## Abstract

Algebra typically represents the students’ first encounter with abstract mathematical reasoning and it therefore causes significant difficulties for students who still reason concretely. The aim of the present study was to investigate the developmental trajectory of the students’ ability to solve simple algebraic equations. 311 participants between the ages of 13 and 17 were given a computerized test of equation rearrangement. Equations consisted of an unknown and two other elements (numbers or letters), and the operations of multiplication/division. The obtained results showed that younger participants are less accurate and slower in solving equations with letters (symbols) than those with numbers. This difference disappeared for older participants (16–17 years), suggesting that they had reached an abstract reasoning level, at least for this simple task. A corresponding conclusion arises from the analysis of their strategies which suggests that younger participants mostly used concrete strategies such as inserting numbers, while older participants typically used more abstract, rule-based strategies. These results indicate that the development of algebraic thinking is a process which unfolds over a long period of time. In agreement with previous research, we can conclude that, on average, children at the age of 15–16 transition from using concrete to abstract strategies while solving the algebra problems addressed within the present study. A better understanding of the timing and speed of students’ transition from concrete arithmetic reasoning to abstract algebraic reasoning might help in designing better curricula and teaching materials that would ease that transition.

## INTRODUCTION

United States National Council of Teachers of Mathematics defines algebra as “a way of thinking and a set of concepts and skills that enable students to generalize, model, and analyze mathematical situations” (National Council of Teachers of Mathematics [NCTM], 2008). This field includes a wide array of topics ranging from elementary linear equation solving to more abstract topics such as modeling given contextual information by formulating complex algebraic expressions. Algebra is usually the first domain in school mathematics that encourages students’ abstract reasoning. By making a transition from concrete arithmetic to the symbolic language of algebra, students develop abstract mathematical cognition essential for their further advancement in mathematics and science. Given that understanding fundamental algebra concepts and acquiring the necessary skills for solving algebra problems requires a certain degree of prior knowledge and abstract thinking, algebra is typically introduced in schools after the development of arithmetic reasoning, as its generalization, usually around the age of 12. This is also roughly the age at which, according to Piaget’s theory of cognitive development, that had a far-reaching influence on both theory and practice in education, a qualitative change in children’s cognitive development occurs (Piaget, 1976). Specifically, this is the age at which most children transition from the concrete operational stage to the formal operational stage (Inhelder and Piaget, 1958; Piaget, 1972). At this time children advance from logical reasoning with concrete to abstract examples, and become able to consider only logical relationships between different elements while ignoring their concrete content. Therefore, this transition from concrete to formal operational stage represents the basis for their further educational advancement. However, many studies have shown that formal reasoning is not developed in most adolescents of that age (Lawson, 1985). Consequently, numerous abstract concepts in mathematics and science curricula are too demanding for the majority of students that remain concrete operational thinkers (Lawson and Renner, 1975). Therefore, it was suggested that teaching abstract concepts should be delayed until the brain maturation permits a transition to the stage of formal operation. Specifically, in the last two decades, brain imaging studies provided new evidence that adolescence represents a period of continued neural development (Blakemore, 2012) that may last longer than would be suggested by Piaget’s theory. In particular, maturational changes in some brain regions that are involved in abstract mathematical reasoning, such as the prefrontal cortex, may last until late adolescence (Giedd and Rapoport, 2010). Educational studies confirm that some tests of prefrontal lobe activity highly correlate with scientific reasoning ability and the capacity to reject scientific misconceptions and adopt correct ideas (Kwon and Lawson, 2000). It seems that children can hardly acquire some abstract reasoning skills until certain age.

In line with arguments suggesting that understanding algebra concepts may be difficult for children in primary schools, research has shown that students indeed often face difficulties in moving from the arithmetic to the algebraic form of reasoning (Kieran, 2004). Despite these findings, many researchers argue for an earlier introduction of algebra in mathematics curriculum (e.g., Carraher et al., 2006; Warren et al., 2006). According to these suggestions, developing algebraic skills and exposing students to more demanding abstract tasks would help in enhancing their abstract reasoning, thus facilitating the transition between cognitive phases. This could be done in a gradual fashion, which is in line with modern mathematics curricula that gradually introduce elements of algebraic thinking in the early grades before formally introducing algebra in the later grades (National Council of Teachers of Mathematics [NCTM], 2000). As an example, since the implementation of a National Curriculum in England, algebra is taught earlier compared to the teaching practice 30 years ago. However, this change of practice has not been overly beneficial, as a recent large-scale survey has shown that the present performance in algebra is broadly comparable to that of students 30 years ago (Hodgen et al., 2010). It seems that the early start of algebra teaching gives an initial advantage to students, which appears not sustained at a later age. Overall, despite many efforts to address students’ difficulties with formal mathematical reasoning, it seems that little advancement has been made (Hodgen et al., 2010).

A more overarching evaluation of students’ success and difficulties in acquiring fundamental algebra concepts is introduced by large international surveys, such as PISA (Program for International Student Assessment) and TIMSS (Trends in International Mathematics and Science Study) that give insights into the quality and efficiency of school systems across many countries. The findings of PISA testing conducted in 2012 with a particular focus on mathematics indicate that students in the highest-performing countries are “more frequently exposed to formal mathematics than students in most of the other PISA-participating countries and economies” (Organisation for Economic Co-operation and Development [OECD], 2013, p. 148). Furthermore, data suggest that the “exposure to more advanced mathematics content, such as algebra and geometry, appears to be related to high performance on the PISA mathematics assessment, even if the causal nature of this relationship cannot be established” (Organisation for Economic Co-operation and Development [OECD], 2013, p. 148). These results indicate a crucial role of algebra in the development of abstract mathematical reasoning.

However, when discussing the acquisition of basic algebra concepts, it is important to highlight that these represent a broad part of school mathematics. As was mentioned earlier, at its fundamental level, algebra includes solving simple algebraic equations that were the focus of the present study. These equations were chosen because equation rearrangement represents a very important skill required for problem solving in many school subjects. Within different teaching frameworks, it is often assumed that, once students learn to solve a simple equations such as, e.g., they can solve such equations for any unknown. This would mean that they are able to solve equivalent simple equations containing both numbers, letters or other symbols. However, physics and chemistry teachers know that students struggle with equations rearrangements, especially for “all-symbol” equations. Küchemann () reported that the majority of the students up to the age of 15 fail to interpret algebraic letters (symbols) as unknowns or generalized numbers, which would be expected from formal operational thinkers. Instead, they still use concrete operational strategies in solving such equations, e.g., ignoring the letters or replacing them with numerical values. This inequivalent treatment of otherwise comparable equations represents only one example of students’ inability to apply the learned principle of equation solving on different instantiations of the same equation format. Given such unequivalences, different mathematics education researchers classify equations in different manners. For example, Usiskin (1988) classifies “equations with letters” used in school algebra as a formula (*A* = *LW*), an equation to solve, (5*x* = 40), an identity (sin *x* = cos *x* tan *x*), a property [1 = *n* (l/*n*)], or a function (*y* = *kx*). Within this, as well as other classifications, it is important to highlight that different types of equations have a different feel not only for students, but also for mathematicians depending on different uses of the idea of a variable (Chazan and Yerushalmy, 2003).

Motivated by these differences, as well as the practical relevance of this topic, the present study was aimed at investigating the developmental trajectory of students’ ability to solve simple algebraic equations. Based on the Usiskin’s (1988) classification, only formulas and equations to solve were chosen, i.e., we used the equivalent 3-terms equations with numbers or with letters. The participants in the study included primary and secondary school students who were all taught equation rearrangement in mathematics at least one year prior to the testing. In addition, they used formulas in other school subjects such as physics and chemistry. However, we hypothesized that, despite repeated exposure and practice with simple algebraic equations, some students of all grades would still struggle with their rearrangement, especially if equations contained only symbols (letters). Furthermore, we were interested in students’ strategies in “all-symbol” equation solving. From our experience and previous studies (Susac et al., 2014, under revision), we assumed that many students use very concrete strategies, such as inserting numbers because it takes time for them to adopt the formal algebraic way of thinking. Consequently, in the present study we explored the age at which the transition from concrete-number-based reasoning to more abstract algebraic reasoning really occurs.

## MATERIALS AND METHODS

### PARTICIPANTS

The participants in the present study included 331 students from five primary and four secondary state schools in Zagreb. With respect to primary school students, all state primary schools in Croatia have the same curriculum, so their students have comparable experiences with algebra education. With respect to secondary schools, we tested students from two gymnasiums (general education and foreign language type schools) and two technical secondary schools. These schools were chosen to represent the average secondary school population in Zagreb mostly preparing for university studies. Specifically, graduates from the two gymnasiums included in the present study typically continue their education at university, typically studying non-mathematics or science related majors. In comparison, graduates from the tested technical schools often continue their education majoring in technical fields. Students from gymnasiums that specialize in natural sciences and mathematics were not included in this study.

The participants in the present study included students from the seventh grade of primary school (age 13–14 years) to the second grade of secondary school (age 16–17 years). Hence, our sample included the students of four age groups, i.e., different school grades: the 7th and 8th grade of primary, and the 1st and 2nd grade of secondary school. Given that in Croatian schools, equation rearrangement is taught at the end of the sixth grade of primary school roughly corresponding to the students’ age of 12–13, all our participants were taught how to solve the task used in the study at least one year prior to this measurement. The number of tested female and male students in each grade is shown in **Table 1**.

**Table 1:** Number of students according to their age and gender.

	7th grade	8th grade	1st grade	2nd grade
Male	36	41	62	54
Female	36	39	33	30
Total	72	80	95	84

The study was approved by the Ethics Committee of the Ministry of Science, Education and Sports, as well as by the schools’ headmasters. Each student’s parents gave an informed written consent before the child took part in the experiment.

### MATERIALS

Raven’s Progressive Matrices were used to assess general cognitive ability (Raven, 1941, 1999). The d2 Test of Attention (Brickenkamp, 1962, 1999) was also administered, but the data were not analyzed in the present study.

A computerized test of equation rearrangement was prepared using E-Prime (Psychology Software Tools Inc., Pittsburgh, PA, USA). In each trial, simple equations consisting of three elements (numbers or letters) were presented in the centre of the visual field. The presented numbers and letters were black, displayed in 24 pt size Ariel font on the white background. Participants’ task was to make *x* the subject of the equation. Simultaneously with the equation, a potentially correct or incorrect answer was presented below the equation. The participants were asked to decide if the offered answer was correct or incorrect.

Three types of equations were used in the study:

A equations: x ⋅ a = b,

B equations: $\frac{x}{a}=b$,

C equations: $\frac{a}{x}=b$.

The offered answers were of the following types: x ⋅ a = b, $\frac{x}{a}=b$, and $\frac{a}{x}=b$. Within all presented equations, *a* and *b* stand for different letters and numbers which all appeared with the same probability during the experiment.

### PROCEDURE

The participants were tested during two school periods (45 min long). During one school period, Raven’s Progressive Matrices and d2 Test of Attention were administered to students in their classrooms. On the same or on another day, students solved the computerized test of equation rearrangement and completed a post-measurement questionnaire in the computer lab.

Before administering the equation rearrangement test, participants were familiarized with the task. They were instructed to respond as quickly as possible by pressing one of the two mouse buttons with their index and middle fingers, corresponding to correct and incorrect answers, respectively. Prior to experimentation, the participants performed a training block consisting of 6 equations equivalent to those used in subsequent experimental trials.

During both practice and experimental trials, each equation was presented until the participant responded, up to a maximum of 30 s. If the participant did not respond within 30 s, the equation disappeared from the screen and another 30 s were available to give an answer. However, these late responses (<0.1% of all trials) were not included in the analysis. After each response, the next equation was presented after a delay of 1 s. Reaction times (RTs) were measured automatically by the computer from the stimulus onset to the participant’s response. No feedback was given to the participants.

During the experiment, the participants were presented with the three previously described types of equations, which were randomized across four blocks. Each block consisted of 15 equations of each equation type, amounting to an overall of 45 presented equations per block. Two blocks contained equations with numbers, while the other two blocks contained equations with letters (symbols). Equations in the first and third blocks contained numbers while those in the second and fourth blocks consisted of letters. The participants could take a break between blocks if needed.

After having finished the computerized test, the participants completed a questionnaire designed for assessing their strategies during equation solving. While responding to these questionnaires, the participants described how they solved each equation type and ranked them by difficulty. In addition, they indicated whether their response depended on the type of the offered answers, and whether they changed their problem solving strategies during the time course of the experiment.

### DATA ANALYSIS

For each participant and each condition, reaction time and accuracy were evaluated. Only correct responses were included in the analysis of RTs. Inverse efficiency was also calculated as the ratio of reaction time and accuracy (Townsend and Ashby, 1978). Lower values on this measure indicate higher efficiency on a particular task. Inverse efficiency is used to account for the speed–accuracy tradeoffs, and we used it as a measure of task difficulty.

To determine the effects of age, gender, level of abstraction, repetitions and equation type, a two-way repeated measures analysis of variance (ANOVA) on accuracy and RTs was conducted. Repeated-measures *post hoc* tests using Bonferroni adjustment were used to further assess the differences between different conditions. In addition, a partial correlation coefficient was calculated in order to determine the relation between participants’ cognitive abilities and their efficacy in equation rearrangement. A threshold of *p* < 0.05 was used for determining the level of effect significance.

To evaluate participants’ strategies in equation solving, we analyzed their answers in the administered *post hoc* questionnaire using the general inductive approach (Thomas, 2003) and descriptive statistical procedures. Each participant’s description of how he/she solved each type of equation was categorized. Hence, different categories reflect different student equation solving strategies, some of which were correct, and some incorrect. Some participants used more than one strategy, and were accordingly assigned to two or more categories. To simplify the comparison of used strategies across participants’ age, all strategies were divided into concrete and rule-based (more abstract) groups. Each participant was assigned to concrete, rule-based or mixed (concrete and rule-based) group. We also evaluated students’ views on equation type difficulty from their ranks provided in the questionnaire.

## RESULTS

### EFFICACY OF EQUATION SOLVING

#### Age and gender effects

**FIGURE 1:**
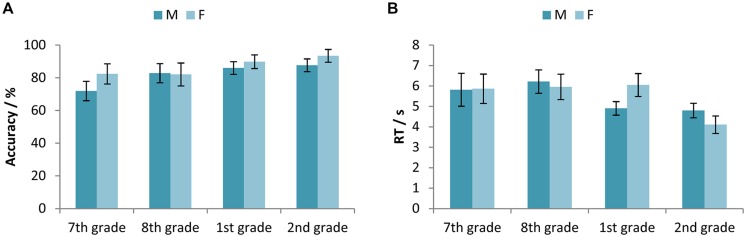
**(A)** Accuracy (percentage of correct responses) and **(B)** RTs for the participants in the 7th and 8th grade of primary school, and the 1st and 2nd grade of secondary school, separated for male and female participants. Error bars represent 95% confidence intervals.

Two-way ANOVAs with factors Age (7th vs. 8th vs. 1st vs. 2nd grade) and Gender (Male vs. Female) were conducted to compare the mean accuracy and RTs. The obtained results for accuracy indicated a statistically significant main effect of Age [*F*(3,323) = 9.43, *p* < 0.001, $\eta_{p}^{2}$ = 0.081] and Gender [*F*(1,323) = 6.40, *p* < 0.05, $\eta_{p}^{2}$ = 0.019], while the interaction effect was not significant [*F*(3,323) = 1.45, *p* > 0.05, $\eta_{p}^{2}$ = 0.013]. **Figure 1A** shows that accuracy increased with the age of participants. On average, girls were more accurate than boys, and the participants in the 7th grade of primary school were less accurate than those in the 1st and 2nd grade of secondary school, while those in the 8th grade were less accurate than the students in the 2nd grade of secondary school.

A corresponding comparison for RTs revealed a statistically significant main effect of Age [*F*(3,322) = 12.91, *p* < 0.001, $\eta_{p}^{2}$ = 0.107] and the interaction effect [*F*(3,322) = 4.14, *p* < 0.01, $\eta_{p}^{2}$ = 0.037]. The main effect of Gender was not statistically significant for RTs [*F*(1,322) = 0.09, *p* > 0.05, $\eta_{p}^{2}$ < 0.0001]. Average RTs decreased with the age of participants (**Figure 1B**). Boys were faster in equation solving in the first grade of secondary school, whereas girls were faster in the second grade.

#### Age and abstraction level effects

**FIGURE 2:**
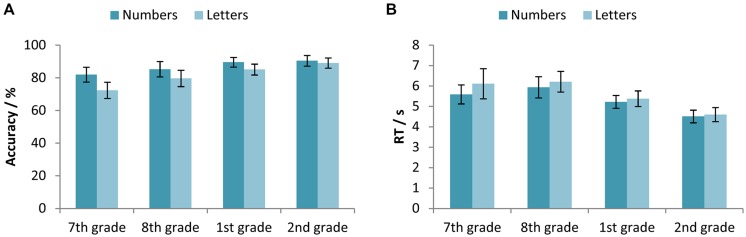
**(A)** Accuracy (percentage of correct responses) and **(B)** RTs for the participants in the 7th and 8th grade of primary school, and the 1st and 2nd grade of secondary school, separated for the equations with numbers and equations with letters. Error bars represent 95% confidence intervals.

To test the differences between participants’ accuracy and RTs in solving equations with numbers and letters across different age, we used the two-way mixed-design ANOVAs with between-subjects factor Age (7th vs. 8th vs. 1st vs. 2nd grade) and within-subjects factor Abstraction level (numbers vs. letters). With respect to accuracy, the statistically significant main effects of Age [*F*(3,327) = 8.37, *p* < 0.001, $\eta_{p}^{2}$ = 0.071] and Abstraction level [*F*(1,327) = 47.17, *p* < 0.001, $\eta_{p}^{2}$ = 0.126], as well as the interaction effect [*F*(3,327) = 4.89, *p* < 0.01, $\eta_{p}^{2}$ = 0.043], were found. Participants were more accurate on equations with numbers, but only in primary school and in the 1st grade of secondary school (**Figure 2A**). In the 2nd grade of secondary school there was no statistically significant difference in the accuracy of solving equations with numbers and letters.

For the RTs, results revealed a statistically significant main effect of both factors, Age [*F*(3,326) = 11.68, *p* < 0.001, $\eta_{p}^{2}$ = 0.097] and Abstraction level [*F*(1,326) = 4.45, *p* < 0.05, $\eta_{p}^{2}$ = 0.013], while the interaction was not significant [*F*(3,326) = 0.61, *p*> 0.05, $\eta_{p}^{2}$ = 0.006]. RTs deceased with age, the participants in the 2nd grade of secondary school were the fastest, and the students in the 1st grade of secondary school were faster than those in the 8th grade. Similar pattern is present in the RTs data as it is in the accuracy data; differences between equations with numbers and letters decreased with the participants’ age (**Figure 2B**).

#### Age and equation type effects

**FIGURE 3:**
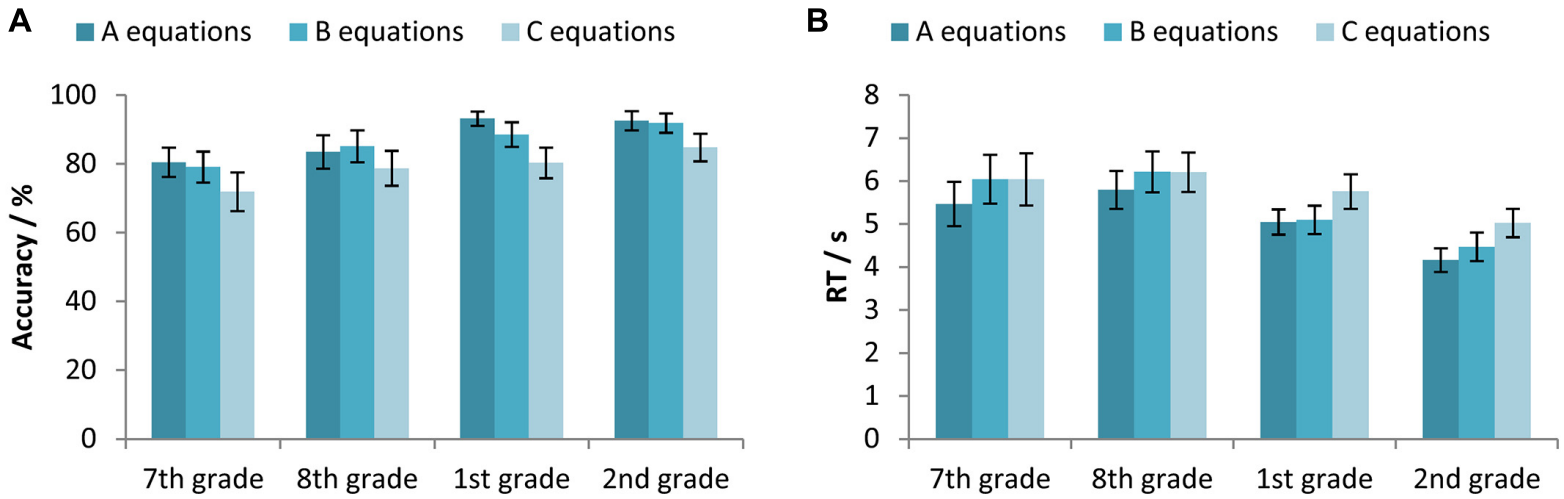
**(A)** Accuracy (percentage of correct responses) and **(B)** RTs for the participants in the 7th and 8th grade of primary school, and the 1st and 2nd grade of secondary school, separated for the different equation types (the A, B and C equations). Error bars represent 95% confidence intervals.

We have used two-way mixed-design ANOVAs with between-subjects factor Age (7th vs. 8th vs. 1st vs. 2nd grade) and within-subjects factor Equation type (A vs. B vs. C equation) to test the differences between participants’ accuracy and RTs for different types of equations across different age. For the accuracy, a significant main effects of both Age [*F*(3,327) = 8.37, *p* < 0.001, $\eta_{p}^{2}$ = 0.071] and Equation type [*F*(2,654) = 66.59, *p* < 0.001, $\eta_{p}^{2}$ = 0.169] were found, as well as their interaction [*F*(6,654) = 2.53, *p* < 0.05, $\eta_{p}^{2}$ = 0.023]. All participants were less accurate on the C equations compared to both the A and B equations, while participants in the 1st grade of secondary school were less accurate on B when compared to A equations (**Figure 3A**).

Corresponding results for the RTs again revealed significant main effects of both Age [*F*(3,326) = 11.68, *p* < 0.001, $\eta_{p}^{2}$ = 0.097] and Equation type [*F*(2,652) = 41.59, *p* < 0.001, $\eta_{p}^{2}$ = 0.113], as well as their interaction [*F*(6,652) = 3.56, *p* < 0.01, $\eta_{p}^{2}$ = 0.032]. Primary school participants solved the A equations faster than the B and C equations, while the secondary school participants were the slowest in solving the C equations. (**Figure 3B**).

#### Age and repetition effects

**FIGURE 4:**
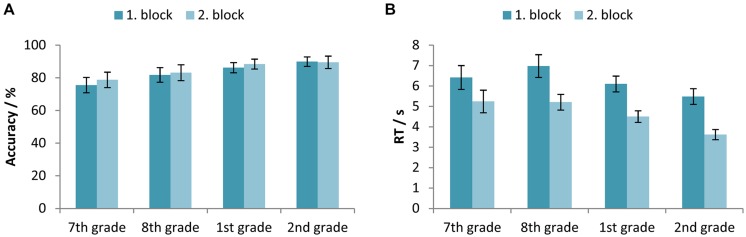
**(A)** Accuracy (percentage of correct responses) and **(B)** RTs for the participants in the 7th and 8th grade of primary school, and the 1st and 2nd grade of secondary school, separated for the first and second block. Error bars represent 95% confidence intervals.

Two-way mixed-design ANOVAs with between-subjects factor Age (7th vs. 8th vs. 1st vs. 2nd grade) and within-subjects factor Block (first vs. second block) were used for testing the differences between participants’ accuracy and RTs across time course of the experiment. The results showed a statistically significant main effect of both factors, Age [*F*(3,327) = 8.37, *p* < 0.001, $\eta_{p}^{2}$ = 0.071] and Block [*F*(1,327) = 5.11, *p* < 0.05, $\eta_{p}^{2}$ = 0.015], while the interaction was not significant [*F*(3,327) = 1.20, *p*> 0.05, $\eta_{p}^{2}$ = 0.011]. **Figure 4A** illustrates a trend of accuracy increase from the 7th grade of primary school until the 1st grade of secondary school, while pairwise comparisons revealed a statistically significant difference between the accuracy levels of participants in the 7th grade when compared to those in the secondary school, and participants in the 8th grade when compared to participants in the 2nd grade of secondary school.

For the RTs, results indicated corresponding significant main effects of both factors, Age [*F*(3,326) = 11.88, *p* < 0.001, $\eta_{p}^{2}$ = 0.099] and Block [*F*(1,326) = 312.27, *p* < 0.001, $\eta_{p}^{2}$ = 0.489], while the interaction was not significant [*F*(3,326) = 2.58, *p*> 0.05, $\eta_{p}^{2}$ = 0.023]. Participants of all ages became faster in equation solving in the second block (**Figure 4B**).

#### Equation solving and cognitive abilities

The relation between the students’ equation solving efficacy and their cognitive abilities was addressed by calculating the partial correlation coefficient between participants’ inverse efficacy and their scores on Raven’s Progressive Matrices score, while controlling for the age effects. The obtained results indicate a statistically significant correlation between equation solving efficacy and cognitive abilities [*r*(307) = –0.22 [95% CI: -0.32, -0.11], *p* < 0.001], indicating that the participants with higher cognitive abilities were generally more efficient in equation solving.

### STRATEGIES USED FOR EQUATION SOLVING

Evaluation of participants’ answers in the questionnaires confirmed that they used different strategies for solving equations with letters. We categorized their answers and divided them into two groups – concrete strategies and rule-based strategies. The most frequently used concrete strategy (37% of all participants) was inserting numbers instead of letters. 11% of participants used a “triangle” memory technique and 4% used a “biggest on the top” strategy that is based on a belief that products and numerators are “big.” For the equation *a*/*x* = *b*, one participant wrote an explanation: “We got *b* by dividing *a* by *x*. Thus, *b* is smallest and *a* is biggest. Then we get *x* by dividing *a* by *b*.”

The most common rule-based strategy (38% of all participants) was a standard application of multiplication/division operations on the equation. 11% of participants reported correctly moving letters to the other side of the equation and often indicated the operation with arrows. The most frequently used incorrect strategy (6%) was to “move letters other than *x* on the other side of equation and change the sign” which meant to change multiplication to division and vice versa. This strategy gave correct responses for the A and B, but not for the C equations. 6% of participants used some kind of a learned rule. For example, one participant wrote for *a*/*x* = *b*: “If *x* is a denominator then the solution is the fraction of the remaining factors, given that the nominator of the initial fraction (the one with *x*) remains the same.” For *a*/*x* = *b* equation (C type), some participants (8%) only swapped *x* and *b* without performing two steps of multiplication and division.

**FIGURE 5:**
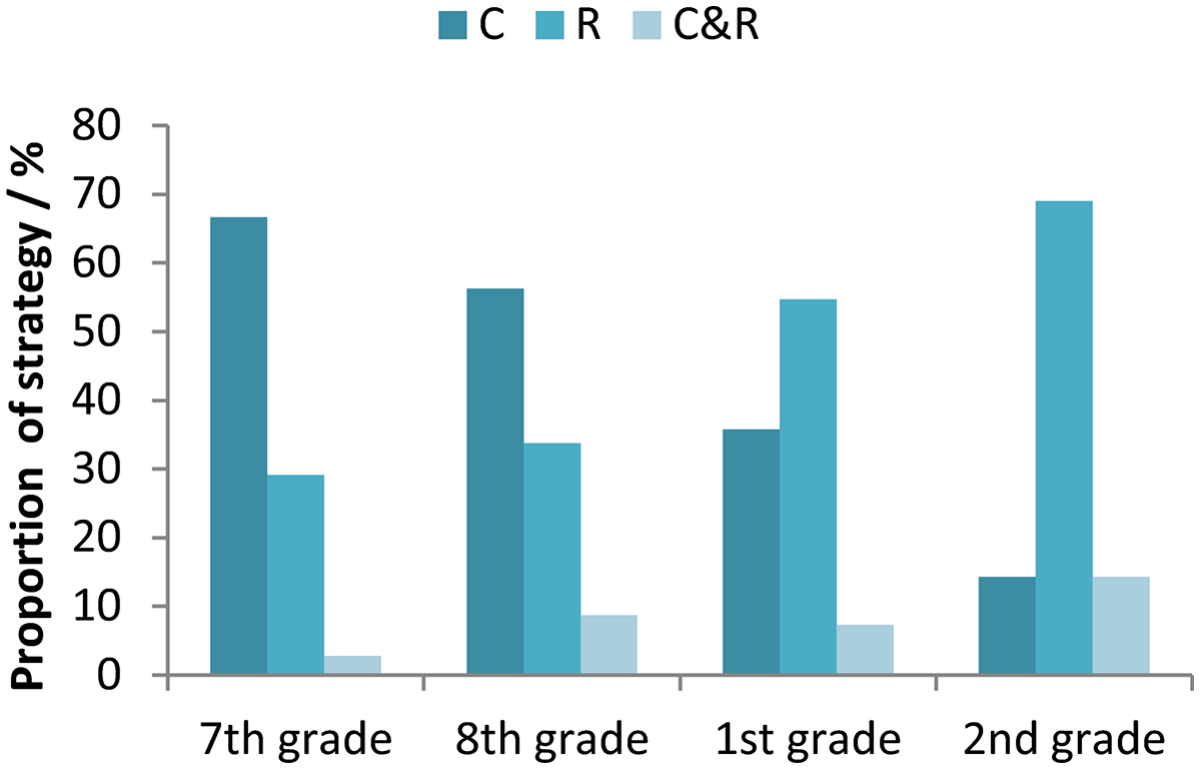
**Proportions of used strategy types (C, concrete; R, rule-based; C&R, concrete and rule-based) for the participants in the 7th and 8th grade of primary school, and the 1st and 2nd grade of secondary school**.

**Figure 5** shows how the proportion of participants who used concrete and rule-based strategies changed with their age. The majority of younger participants (from primary school) used concrete strategies, whereas participants from secondary school mostly used more abstract, rule-based strategies. Some participants used both concrete and rule-based strategies. For example, one participant used standard multiplication/division procedure for the A and B equations, but she inserted “real numbers” to solve the C equations. Participants who used both concrete and rule-based strategies typically used a concrete strategy to solve the C equations.

### EQUATION DIFFICULTY RANKS

**FIGURE 6:**
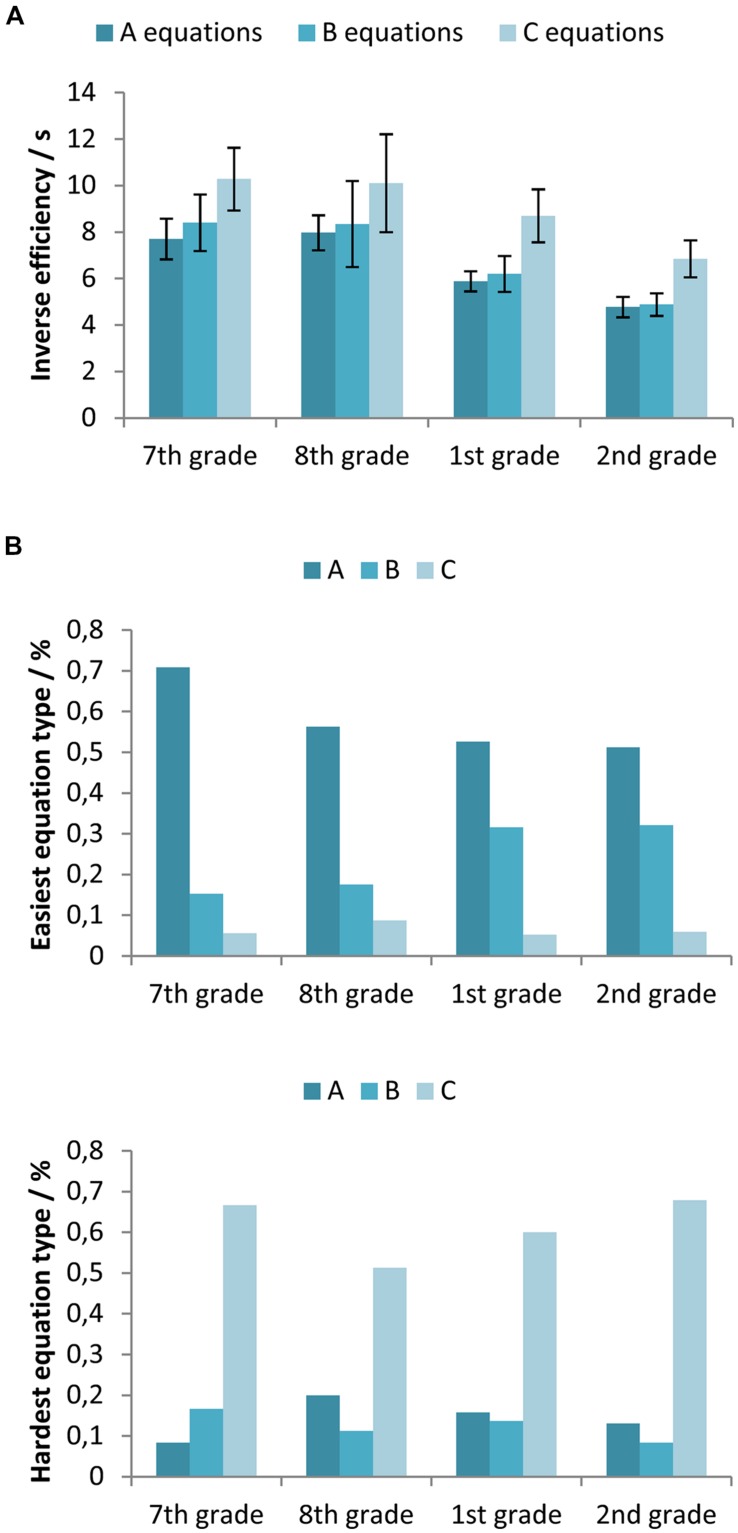
**(A)** Inverse efficiency on equations with letters for the participants in the 7th and 8th grade of primary school, and the 1st and 2nd grade of secondary school, separated for the different equation types (the A, B, and C equations). Error bars represent 95% confidence intervals. **(B)** Proportion of equation types ranked as the least difficult and the most difficult by the participants in each grade.

**Figure 6A** shows the inverse efficiency measures for different equation types (all with letters) across different participants’ age. To test the differences between participants’ inverse efficiency in solving different types of equations a two-way mixed-design ANOVA with between-subjects factor Age (7th vs. 8th vs. 1st vs. 2nd grade) and within-subjects factor Equation type (A vs. B vs. C equation) was used. The obtained results indicate a statistically significant main effect of both factors, Age [*F*(3,325) = 11.84, *p* < 0.001, $\eta_{p}^{2}$ = 0.099] and Equation type [*F*(2,650) = 43.72, *p* < 0.05, $\eta_{p}^{2}$ = 0.119], while the interaction was not significant [*F*(6, 650) = 0.33, *p*> 0.05, $\eta_{p}^{2}$ = 0.003]. If we adopt inverse efficiency as a measure of task difficulty (Townsend and Ashby, 1978), the results suggest that the C equations were the most difficult. There was no statistically significant difference between the A and the B equations.

Participants ranked different equation types by difficulty in the questionnaires. 28 participants reported that all equation types are equally difficult. Three participants thought that equations with multiplications (A type) are easier than equations with division (B and C). Eight participants did not provide an answer to this question. **Figure 6B** shows the data of the remaining participants across their age groups. Most participants reported that the A equations were the easiest. However, a considerable number of the secondary school students (32%) thought that the B equations were the easiest. Most participants agreed that the C equations were the most difficult.

## DISCUSSION

### ACCURACY AND SPEED OF EQUATION SOLVING

The results obtained in the present study suggest that the tested students were overall rather successful in equation rearrangement, with accuracy levels amounting to an average of 85%. Although this may seem quite high, if the true-false nature of test items is taken into account this becomes a less satisfactory result, especially for all-symbol equations which were correctly solved by 82% of the participants. However, our data indicate that students become more efficient, i.e., more accurate and faster, in higher school grades.

With respect to gender differences, the girls in our sample were on average more accurate in equation rearrangement than boys, while no significant differences in their speed were revealed. This finding is in disagreement with a common belief that boys are better in mathematics than girls which is based on reports that boys outperform girls on standardized tests like SAT (e.g., Byrnes and Takahira, 1993). However, most studies report no differences between boys and girls on algebra assessments (e.g., Bridgeman and Wendler, 1991). In fact, girls sometimes do even better than boys (e.g., Else-Quest et al., 2010), while male superiority among adolescents is usually related to boys’ spatial reasoning and more diverse strategies in problem solving (Geary, 1996). In the present study we observed slightly higher accuracy for girls than for boys, but overall comparable speed of equation solving, which resonates with previous findings suggesting small, if any, gender differences in solving simple algebraic equations.

It is important to emphasize that students’ success in solving simple algebra equations differed across different types of equations. Specifically, within the present study we compared equivalent equation formats that contained either symbols or numbers. As expected, the obtained results indicate that the younger participants were more accurate and faster in solving equations with numbers than with letters although these were equivalent. This indicates that younger students still struggle with more abstract equations. In contrast, students in the 2nd grade (age 16–17 years) had a comparable level of accuracy and RTs for equations with numbers and letters. This indicates that they reached an adequate level of formal reasoning (Inhelder and Piaget, 1958), at least for this particular task.

Next, we compared participants’ efficacy in solving three different types of equations. The lowest accuracy and the longest RTs obtained for the C equations (*a*/*x* = *b*) suggest that this was the most difficult type of equation. Younger students were struggling with this equation type; the accuracy of the 7th grade participants (age 13–14 years) was only 72%. Accuracy on the C equations increased with the participants’ age, with 2nd graders (age 16–17 years) reaching 85%. These results reflect the fact that two operations are needed to solve C equations, and only one operation for other two types of equation, thus indicating that the procedural complexity has also a significant effect on efficiency in equation solving. Our data suggest that even our oldest participants, 16–17 years old at the time of the testing, had difficulties with the slightly more difficult, but still very simple equations. This is in agreement with the previous reports on students’ difficulties with “all-symbol” equations (Ekenstam and Nilsson, 1979; Küchemann, 1981).

In addition to exploring age, gender and equation type effects, within the present study we also explored practice effects across all equation types. Our participants became faster and more accurate in equation rearrangement during the time course of the measurement. This finding is in agreement with a previous report indicating how children become faster during a 5-day practice in algebra equation solving (Qin et al., 2004). It seems that some of our participants learned how to solve equations as they were repeatedly exposed to them for a short period of time, even without feedback. Even the participants from the 2nd grade of secondary school (age 16–17 years), who had stable high accuracy levels from the beginning until the end of the measurement, became faster in equation rearrangement. This might be an interesting finding for mathematics teachers. However, additional studies are needed to explore a long-term effect of such short and intense practice in equation solving.

Furthermore, our results showed that the participants with higher cognitive abilities were more efficient in equation solving. This is in line with the previous longitudinal testing which indicated that students with higher IQ scores tended to demonstrate higher cognitive levels and made faster progress through algebra levels than students with lower IQ scores (Küchemann, 1981). It has been suggested that on familiar algebra tasks, participants rely on automated routines and acquired facts that are more systematically learned by individuals of higher cognitive abilities (Bornemann et al., 2010). Consequently, they outperform individuals with lower general cognitive abilities, while allocating the same amount, or even less, of cognitive resources to the task. Accordingly, we could conclude that our participants with higher abilities profited from more efficient processes compared to individuals of lower cognitive abilities. However, the general cognitive ability is not the only factor influencing individual’s understanding of algebraic equations. Other factors are also important, such as the intuitive assumptions and pragmatic reasoning about a new notation, analogies with familiar symbol systems, interference from new learning in mathematics, and the effects of misleading teaching materials (MacGregor and Stacey, 1997).

### CONCRETE AND RULE-BASED STRATEGIES FOR EQUATION SOLVING

Half of the participants used concrete strategies for equation rearrangement and the most frequently used concrete strategy was inserting numbers into equations. When using this strategy, the students think of an equivalent equation with numbers, solve it and then apply the solving algorithm on the equation with symbols. For example, for the A equation (*x* ⋅*a* = *b*), they insert numbers so the equation becomes 2⋅3 = 6 and then conclude that “if 2 = 6/3, then *x* = *b*/*a*.” It seems that these participants have not yet reached the formal operational stage and are more comfortable with concrete numbers in equations. This is in agreement with the previous studies on algebraic processing in adolescents (Ekenstam and Nilsson, 1979; Küchemann, 1981; Susac et al., under revision).

A considerable number of participants (11%) used the “triangle” method often taught by physics teachers to “simplify” equation rearrangement for their students. Within this strategy, a triangle is divided into three parts. Two quantities that are multiplied together are written side-by-side at the bottom of the triangle. The remaining quantity (their product) is written at the top. For *x* ⋅*a* = *b* (A equation), *x* and *a* are written at the bottom, and *b* at the top. If we want to make *x* subject of the equation, *x* should be covered and what is left, namely “*b* over *a*,” represents the result. Although this strategy helps students in equation rearrangement, this technique does not develop their formal reasoning.

The “biggest on the top” strategy also has origin in concrete way of thinking. As few participants reported, they always considered product in multiplication equations and numerator in division equations as the biggest object that helped them in the rearrangement. For example, in the A equations (*x* ⋅*a* = *b*) they regard *b* as the biggest object that helped them to form a solution (the biggest goes on the top, therefore *x* = *b*/*a*). Although they did not explicitly insert numbers into equations, participants’ experience with natural numbers may probably account for their line of reasoning (the biggest number is always the product of two natural numbers, and the numerator is bigger than the denominator and the result of a division). In our previous study we have found that the UK students also use this strategy (Susac et al., under revision).

More than half of the participants (56%) were reasoning more abstractly while solving at least one equation type, i.e., they were applying rules. During the testing, few participants made a transition from concrete substitution of letters by numbers to the recognition of patterns and rules. The most frequently used rule-based strategy was multiplication and division of equation with the “letter next to *x*.” This procedure was performed correctly by the majority of participants who decided to use it. However, the most common incorrect strategy involved the procedure of moving “letter next to *x* ” on the other side of equation and changing the operation, multiplication to division and vice versa. This probably reflects an inappropriate application of the procedure learned for equations with addition/subtraction, indicating that the application of mathematical rules and procedures can be very confusing for students.

As in our previous study (Susac et al., under revision), some participants reported moving letters to the other side of equation. This corroborates findings showing that spatial reasoning is closely related to the number sense (as in the case of mental number line; e.g., Dehaene, 1997) and mathematical operations in general. A number of neuroimaging and neuropsychology studies have demonstrated that the relationship between number and space processing is deeply rooted in the organization of parietal circuits for these capacities (Hubbard et al., 2005). Mathematical experts in our previous study often used spatial terms when explaining their strategies in equation solving (Susac et al., under revision). It seems that the development of spatial reasoning in students might be beneficial even in “non-spatial” areas of mathematics such as algebra. In addition, visualization can be also helpful in developing problem-solving skills in mathematics (Scheiter et al., 2010)

Some participants reported strategies based on some types of rules that they developed by themselves. By repeated exposure to equation rearrangement, they recognized some patterns from which they derived some general rules. Although participants’ rules were not always correct, they possibly represent a step in developing more consistent and correct solving strategies. A number of participants recognized that they do not have to perform two steps of multiplication and division for *a*/*x* = *b* equation (C type), and just swapped *x* and *b*. In doing so, they developed a new, more efficient strategy during the experiment, through pattern recognition which is of great value in performing algebraic tasks (Orton and Orton, 1999).

Overall, the obtained results suggest that the proportion of concrete strategy usage decreases at the same time as the proportion of rule-based strategies increases with the age of participants. This progression is gradual and it probably continues after the 2nd grade of secondary school (age 16–17 years). Our data confirm that the development of algebraic thinking is a process which unfolds over a long time. Consequently, we can conclude that children at the age 14–15 are in transition from concrete to abstract strategies in algebra that is in agreement with previous research (Küchemann, 1981).

### EQUATION DIFFICULTY RANKS

To determine the difficulty of different types of equations with letters, we evaluated inverse efficiency across the age groups of our participants. In all age groups, participants were the least efficient in solving the C equations, which suggests that these are the most difficult equation types. This finding was expected, because the C equations are usually solved in two steps while only one step is needed for the A and B equations. Not all participants performed two operations in solving *a*/*x* = *b* (e.g., some of them swapped *x* and *b*). However, for C equations, both equation and solution involve division, which is generally more difficult than multiplication (Hecht et al., 2003).

Inverse efficiency measures indicated that the A equations were of similar difficulty as the B equations. The B equations, *x*/*a* = *b*, are probably the easiest because their solution is based on multiplication and the order of the variables in the product is not important. In the A equations, *x* ⋅*a* = *b*, the solution includes a division so an additional step to decide the right order of numerator and denominator is needed (as *a*/*b* is not the same as *b*/*a*). However, it seems that our participants were not fully aware of this pattern, as can be observed in their inverse efficiency results.

It is interesting to note that a large majority of participants reported that the B equations are more difficult than the A equations although this is not supported by the obtained results. Probably their self reports were again influenced by the fact that division is perceived as more difficult than multiplication. However, in judging equation difficulty, participants failed to take into account the fact that correctly solving these equations also includes these operations. Still, the increased number of participants who ranked B equations as easiest among older students suggests that some older participants (from secondary school) became aware of the patterns in the task. In addition, it seems that metacognitive skills improve with age as secondary school students, on average, ranked equation difficulty more accurately than younger participants. This finding concurs the previous reports on the importance of metacognitive activities for success in problem solving in mathematics (Kramarski and Mevarech, 2003; Cohors-Fresenborg et al., 2010).

## CONCLUSION

The goal of the present study was to investigate the development of students’ abstract reasoning skills on a simple equation rearrangement task. Although all our participants learned equation rearrangement in mathematics at least one year prior to our testing, and were required to solve simple equations in mathematics and science problems, they still had difficulties with some equation types. However, accuracy and speed of equation rearrangement increased with the participants’ age. Younger participants were more accurate and faster in solving equations with numbers than with letters, suggesting that they are still concrete thinkers. The difference in the efficacy of solving equations with numbers and letters disappeared for participants from the 2nd grade of secondary school (age 16–17 years), indicating their ability to think more abstractly, at least on our task. The transition from concrete to formal reasoning was also reflected in strategies that the participants used for solving equation with letters. Younger participants from the primary school (age 13–15 years) mostly employed concrete strategies such as inserting numbers, while secondary school participants (age 15–17 years) mainly used rule-based strategies.

Our results indicate that the transition from concrete to abstract reasoning represents quite a long process, even for simple algebraic task used in this study. Teachers and educational policy makers should be aware that it is not enough to learn about equation rearrangement in mathematics once. It should not be presumed that students master this skill quickly and that they can easily apply it in other context such as problem solving in physics. On the contrary, teachers should use every opportunity to encourage students to use formal reasoning – both pattern recognition and effective application of mathematical rules and known procedures.

## Conflict of Interest Statement

The authors declare that the research was conducted in the absence of any commercial or financial relationships that could be construed as a potential conflict of interest.
